# Influences on improved confidence among allied health students in working with Australian Indigenous people during a rural placement: a pre-post survey study

**DOI:** 10.1186/s12909-024-06207-2

**Published:** 2024-11-01

**Authors:** John A. Woods, Kathryn Fitzgerald, Lennelle P. Papertalk, Charmaine Green, Rohan L. Rasiah, Monica Moran, Samantha Bentink, Sandra C. Thompson

**Affiliations:** Western Australian Centre for Rural Health, School of Allied Health, The University of Western Australia (M315), 35 Stirling Highway, Perth, WA 6009 Australia

**Keywords:** Health workforce, Australian Aboriginal and Torres Strait Islander Peoples, Rural health services, Education, Professional, Allied health occupations

## Abstract

**Background:**

Together with addressing social determinants of health, culturally safe healthcare provision is essential for closing the health outcomes gap experienced by Aboriginal and Torres Strait Islander (Indigenous) Australians. Rural placements potentially provide students of the health professions with opportunities to enhance their knowledge and skills regarding cultural safety. We used rural placements data systematically collected from allied health students, including commencement- and end-of-placement questionnaire responses, to investigate the determinants of confidence in working with Indigenous people.

**Methods:**

The study comprised data from all students who provided survey data at both commencement and end of their first placement directly supervised by the administering University Department of Rural Health during the period 2019–2022. Five-point ordered responses to the question ‘How confident do you feel about working with Aboriginal people?’ were used to assess student and placement-related determinants of confidence (Confident/Very confident versus other) at baseline and increased confidence (≥ 1 point) during the placement using crude and adjusted multivariable robust Poisson regression.

**Results:**

Participating students (*N* = 489) were from diverse allied health disciplines (including pharmacy *n* = 94, 19.2%; chiropractic *n*= 66, 13.5%; physiotherapy *n*= 65, 13.3%; social work *n* = 59, 12.1%; and occupational therapy 58, 11.9%). Confidence in dealing with Aboriginal people was lower at commencement among females compared with males (adjusted relative risk [aRR] 0.65; 95% confidence interval [CI] 0.53–0.80), and higher among students of Australian rural origin compared with others (aRR 1.49; CI 1.22–1.83) and those who reported previous experience working with Indigenous people compared with those reporting none (aRR 1.40; CI 1.14–1.72). Placement attributes associated with increased confidence working with Indigenous people between placement commencement and end were interaction with Indigenous people within the placement (aRR 2.32; CI 1.24–4.34), placement model reflecting more structured academic supervision (aRR 1.18; CI 1.02–1.37), and placement length (aRR per additional day 1.002; CI 1.001–1.004). These associations were robust to modelling that accounted for a ceiling effect on increased confidence.

**Conclusions:**

While influenced by students’ demographic attributes and prior experiences, confidence of allied health students in working with Indigenous people is enhanced during rural placements, particularly through direct contact with Indigenous people.

**Supplementary Information:**

The online version contains supplementary material available at 10.1186/s12909-024-06207-2.

## Background

Healthcare in Australia, underpinned by a universal insurance system (Medicare), is widely considered to be among the world’s best [1, 2]. However, there are areas of inequity in service provision, with rural and remote areas often underserved and characterised by high turnover of health workers [3, 4]. Moreover, Aboriginal and Torres Strait Islander (henceforth Indigenous) people in Australia experience health outcomes less favourable than those of their non-Indigenous counterparts, with a substantial (> 8 years) life-expectancy deficit in the Indigenous population [5, 6]. This outcome gap is multifactorial, and substantially attributable to social and cultural determinants of health [7] consequent to colonial settlement and associated violence, discrimination, and intergenerational trauma [8, 9]. It also reflects health system failures including structural racism [10] and the underservicing of non-metropolitan areas, particularly more remote areas where many Indigenous people reside.

Improved servicing of rural and remote areas requires augmenting the rural health workforce through increased recruitment and retention of health professionals. A key strategy for developing and sustaining a rural health workforce is encouragement of students in the ‘pipeline’ of the health professions [11]. This includes exposure to rural and remote practice through practicum placements in professional degree programs [12–15] and continues through opportunities for rural employment in the years immediately following graduation [16]. Nowadays, many Australian health profession students have the opportunity to participate in rural health placements [17].

Typically, among the objectives of rural placements is creating opportunities to enhance students’ cultural understanding and ability to communicate and work with Indigenous Australians [18]. Although there is uncertainty concerning the influence of attitudes among health professionals such as their confidence working with Indigenous people on their provision of culturally safe care [19], there is evidence that lack of confidence may reduce service quality in this regard [20]. However, there is scant evidence on the determinants of efficacy in rural placements for improving students’ confidence in this regard.

One of nineteen University Departments of Rural Health currently existing nationwide, the Western Australian Centre for Rural Health (WACRH), established in 1998, provides rural placements for undergraduate and postgraduate students of the health professions from multiple institutions. WACRH has its main office based in Geraldton in the Midwest region and another major office based in the more remote Pilbara region of Western Australia.

Using data systematically collected from allied health students undertaking placements with WACRH, including responses to questionnaires from the commencement and end of placement, the aims of the current study were to investigate the influence of student attributes and experiences during the placement on developing confidence in working with Indigenous people.

## Methods

### Student placements

Student rural placements administered by WACRH are undertaken in diverse healthcare settings, including domestic and residential aged-care services, schools and playgroups, and Aboriginal Medical Services. Each placement is based on one of several different models for academic and vocational supervision WACRH has developed a classification system for rural student placements based on the extent to which academic staff are directly involved with coordinating, hosting, supervising the placement and assessing the student’s performance. Accordingly, WACRH involvement in placements is categorised as: Comprehensive, Blended, Supported/Liaison, or Assisted (Table 1).

**Table 1:** Western Australian Centre for Rural Health (WACRH) Student Placement Models

Placement type	Description	Assessment
Comprehensive	Students coordinated, hosted, supervised and assessed by WACRH staff.	WACRH supervisor
Blended	Students hosted, coordinated and primary supervision provided by WACRH. They may have a small *part* of their clinical/ fieldwork activities directly supervised from an external supervisor, however the responsibility for placement and clinical fieldwork experience on a day-to-day basis is with the primary WACRH supervisor.	WACRH supervisor responsible for assessment. External supervisor may have some input
Supported/Liaison	Students placed with an external agency for day-to-day clinical placement/fieldwork with on site, same-discipline supervision. WACRH may add value to placements e.g., coordination, liaison, cultural orientation or other clinics or other activities.	An external supervisor is responsible for completing the student assessment. WACRH supervisor may have liaison/ support role in assessment
Assisted	Students supported to participate in their rural placement by WACRH but not be directly involved with WACRH staff or programs, though they may be provided accommodation by WACRH.	Externally assessed. No WACRH involvement

The placements incorporate orientation to Indigenous culture, Indigenous ways of knowing, and Indigenous health issues through modules and workshops that have been developed and led by Indigenous staff. Firstly, students undertaking a placement are recommended/required to complete an online Indigenous cultural orientation training module prior to commencement. WACRH provides face-to-face cultural orientation at the commencement of placements at most sites. For example, the three-hour Aboriginal Miyarnuwimanha Cultural Orientation is recommended for students early in their placement commencement in Geraldton where it is delivered by the local Indigenous staff who developed it. ‘Miyarnuwimanha’ translates to ‘learning’ or ‘becoming knowledgeable’ in the language of the local Wajarri Yamaji people. During the study period, several different approaches to cultural orientation have been provided for students based in the Pilbara.

### Design, data source and sample

WACRH has systematically collected data from all students undertaking rural placements for many years, with substantial ongoing refinements to data collection from 2017. The records used in this study were those of all rural placements that (i) were identifiably a student’s first placement coordinated by WACRH (ascertained by lookback to 2013), (ii) were undertaken in the period 01/01/2019–31/12/2022 (because all relevant variables were collected only from 2019 onwards), (iii) were among students who identified both as non-Indigenous and a citizen or permanent resident of Australia, (iv) had provided relevant questionnaire data at both the commencement and the end of their placement, and (v) had no missing values for other study measures (below).

### Study measures: (i) outcome variable

All students who take a placement with WACRH are encouraged to participate in an online questionnaire at both the commencement and end of the placement. The questionnaire at both time points includes items with a five-point ordered response agreement scale in relation to the student’s confidence in dealing with several aspects of a rural placement. Among these items, students are asked, ‘How confident do you feel about working with Aboriginal people?’ The outcome measure for this study indicated the change in a student’s confidence working with Indigenous people between the commencement and end of their rural placement. The five-point ordered response to this item was firstly collapsed into binary form (‘Confident’ or ‘Very Confident’ versus ‘Not at all confident’ or ‘Not confident’ Or ‘Neutral’). Secondly, a single-point or greater increase in confidence between the commencement and end questionnaires was considered ‘increased confidence’. In 2022, the distribution of end-of-placement questionnaires was disrupted by a systems problem related to staff turnover, which was not rapidly identified, markedly reducing the proportion of students who completed the survey during that year compared with the previous years of the study.

### Study measures: (ii) other covariates

Detailed data on the demographic and educational characteristics of all students undertaking rural placements with WACRH are collected routinely at registration prior to placement commencement. These data include the student’s date of birth, from which age in years at placement commencement was calculated. Age was collapsed into binary groups (< 23 years versus ≥ 23 years) for the analysis in consequence of non-linearity in the relationship between age and the outcome variable detected during preliminary regression modelling. Other student characteristics included as covariates in the current study were gender (female, male or other); Aboriginal and/or Torres Strait Islander identification; Australian citizenship/permanent residency status; rural origin (defined dichotomously as having or not having ‘lived in a rural area for a total of ten years or for five consecutive years’); and current educational enrolment (including institution, professional discipline of study, undergraduate versus postgraduate level of the degree, and international student enrolment status). An additional item from the placement registration process included as a study covariate was the question, ‘Do you have experience working or interacting with Aboriginal and/or Torres Strait Islander peoples?’

Placement length in days, calculated arithmetically from commencement and end dates, was demonstrated to be linearly related to the outcome and was modelled as a continuous variable.

The end-of-placement questionnaire also includes the two questions, ‘During your placement, did you have the opportunity to work or interact with Aboriginal people as clients, co-workers or community members during the clinical/placement practice?’ and ‘During your placement, did you have the opportunity to work or interact with Aboriginal people as clients, co-workers or community members outside of the clinical/placement practice?’

In consideration that the placement model may have influenced students’ experiences during the placement and thereby their responses to end-of-placement questionnaire responses, Placement type was modelled as a covariate in this study and was collapsed into binary form for analysis (Comprehensive or Blended versus Supported/Liaison or Assisted).

Finally, the calendar year in which the placement was undertaken was included as a covariate in order to investigate any change over time and specifically to determine any influence of the COVID pandemic (i.e., through comparison of outcomes during each year from 2020 onwards in comparison with the pre-pandemic year of 2019).

### Statistical analysis

Baseline demographic and educational characteristics of student participants were quantified descriptively. Moreover, in order to assess the potential for bias arising from the unavoidable restriction of the study sample to students who completed questionnaires at both the commencement and end of their placement, a descriptive comparison was made of the baseline characteristics of (i) all students who participated in the commencement-of-placement questionnaire, and (ii) the subgroup of these who comprised the study sample (i.e., who participated in both commencement and end questionnaires and provided paired commencement/end-of-placement data on confidence working with Aboriginal people).

The proportions of students (i) in the overall sample, and (ii) in selected subgroups who reported feeling ‘Confident’ or ‘Very Confident’ about working with Aboriginal people at the commencement and end of placement were calculated and presented graphically.

The outcome variable was collapsed into binary form for analysis because the five-point scale was not suitable for modelling as a continuous variable, and modelling of change in confidence in ordinal form was not appropriate because the data did not conform to the proportional odds assumption that underlies ordered logistic regression (at least in relation to the traditional method that provides readily interpretable single estimates for each covariate association) [21]. Considering that odds ratio inflate intuitive between-group differences when outcomes are common [22], we opted to determine relative risk directly using robust Poisson regression models [23] to estimate (i) crude and mutually adjusted influences of student baseline demographics and placement characteristics on feeling ‘Confident’ or ‘Very Confident’ at placement commencement, and (ii) of reporting increased confidence at end compared with commencement of placement. Considering the potential for a ‘ceiling effect’ in increased confidence due to a small proportion of students recording the maximal level of confidence in the commencement survey, we performed a sensitivity analysis from which these individuals were excluded. All data analyses were performed using Stata® Version 16 (Stata Corporation, College Station, Texas, USA). Statistical significance was set at the 5% level.

### Ethics

The University of Western Australia’s Human Research Ethics Committee Ethics provided approval for the study (reference 2023/ET000289), including the justification of waiver for student participants’ informed consent. Two of the authors (LP and CG) are local Indigenous staff raised in the Midwest who deliver cultural learning opportunities for students based in Geraldton.

## Results

**Fig. 1:**
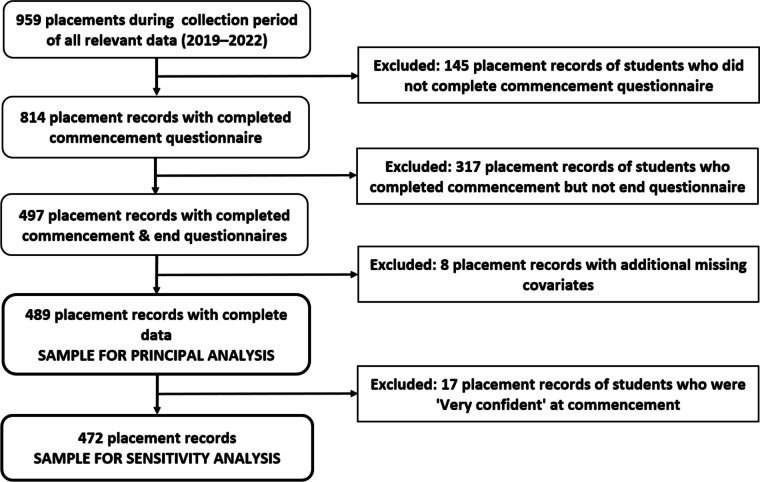
Placement record selection procedure

Among 959 students undertaking their first placements co-ordinated by WACRH during the years 2019–2022, 489 were eligible for inclusion in the study (Fig. 1). Non-completion of surveys impacted on the sample of eligible students, with 145 otherwise eligible students (15.1%) failing to complete the commencement survey, followed by substantial further attrition due to 317 (38.9%) of the remaining 814 students not completing the end-of-placement survey. Non-completion of end-of-placement surveys was especially pronounced in the 2022 student cohort (72.6% of those who had provided a commencement survey, compared with 23.1%–35.8% in the previous years, i.e., 2019–2021 [Table S1]).

The students in the sample were demographically and educationally diverse (Table 2). They represented a broad range of allied health disciplines across both undergraduate (*n* = 329, 67.3%) and postgraduate (*n* = 160; 32.7%) professional degree programs, with the majority enrolled in pharmacy (*n* = 94, 19.2%); chiropractic (*n* = 66, 13.5%); physiotherapy (*n* = 65, 13.3%); social work (*n* = 59, 12.1%); and occupational therapy (58, 11.9%).

**Table 2:** Baseline characteristics of allied health students (*N* = 489) and their rural placements

	**Range**	**Median**	**Mean**	**SD**
**Age (years)**	18–60	23	25.1	6.0
			**n**	**%**
**Gender**	Female		349	71.4
	Male		140	28.6
	Other		0	0.0
**Rural origin**	No		375	76.7
	Yes (Australia)		108	22.1
	Yes (Overseas)		6	1.2
**Qualification level**	Undergraduate		329	67.3
	Postgraduate		160	32.7
**Prior Indigenous experience**	No		323	66.1
	Yes		166	33.9
**Placement model**	Assisted/Supported		272	55.6
	Comprehensive/Blended		217	44.4
**Placement year**	2019		139	28.4
	2020		122	25.0
	2021		176	36.0
	2022		52	10.6
**Discipline of study**^**a**^	Audiology		11	2.3
	Biomed/Med Science		< 5	< 1.0
	Chiropractic		66	13.5
	Dietetics/Nutrition		14	2.9
	Exercise Physiology		38	7.8
	Health Promotion		13	2.7
	Health Science		< 5	< 1.0
	Medical Imaging		15	3.1
	Music Therapy		< 5	< 1.0
	Occupational Therapy		58	11.9
	Pharmacy		94	19.2
	Physiotherapy		65	13.3
	Podiatry		24	4.9
	Public Health		6	1.2
	Social Work		59	12.1
	Speech Pathology		22	4.5

^a^Discipline of student presented to demonstrate diversity of sample but not included as a covariate in analyses. Cell numbers < 5 obscured to protect confidentiality

*SD *Standard deviation

**Fig. 2:**
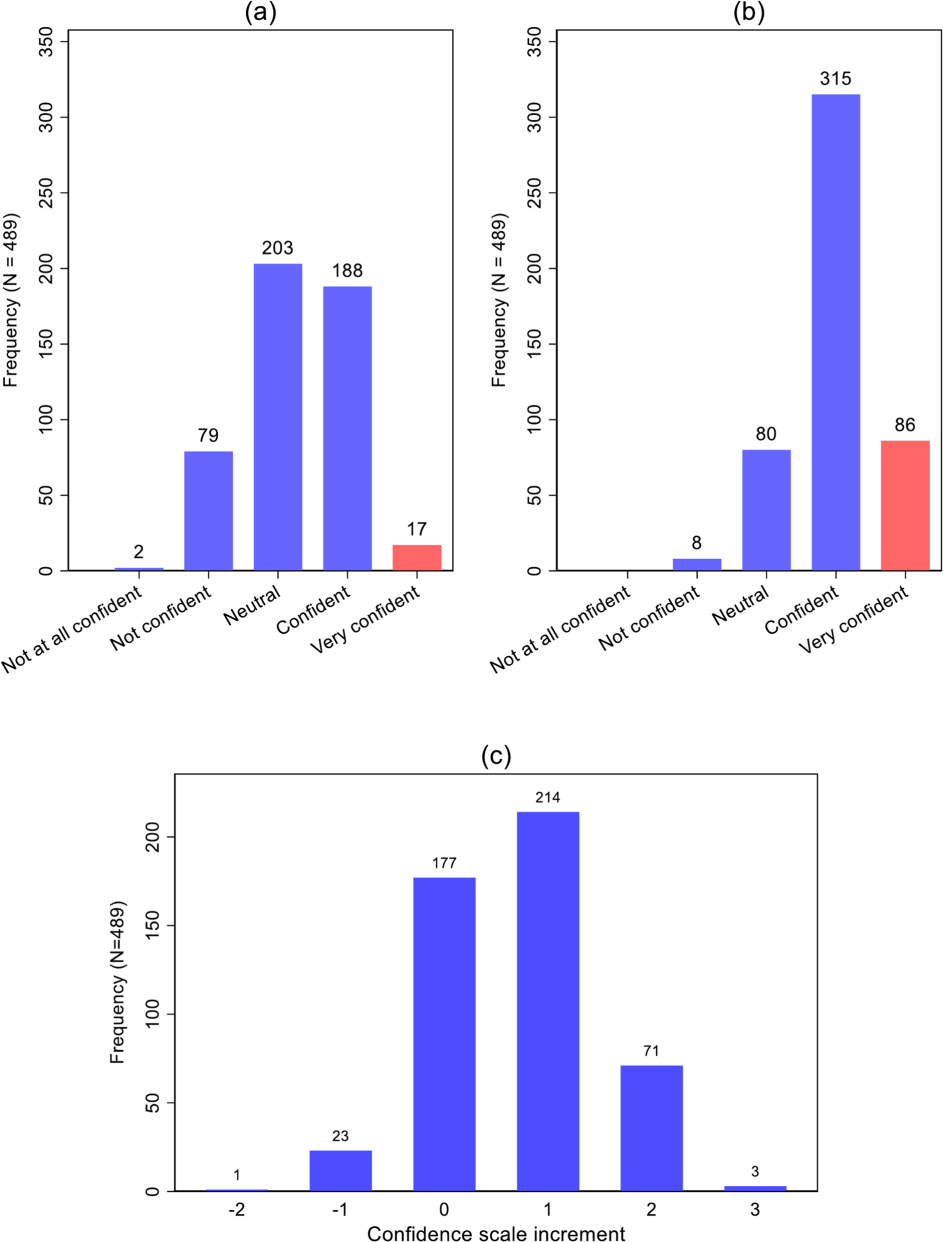
**a** Distribution of student responses to five-point item on confidence working with Aboriginal people at commencement of placement (maximum possible responses highlighted); **b** Distribution of student responses to five-point item on confidence working with Aboriginal people at end of placement (maximum score highlighted); **c** Distribution of changes in confidence between commencement and end of placement

In the placement commencement survey, 17 students (3.5%) reported the highest possible score for confidence working with Aboriginal people (‘Very confident’), and were therefore not able to enter an end-of-placement response that indicated an increase in confidence during the placement (Fig. 2). Feeling ‘Confident’ or ‘Very confident’ working with Aboriginal people at the commencement of the placement (Table 3) was independently associated with gender (adjusted relative risk [aRR] for female compared with male students 0.65; 95% confidence interval [95% CI] 0.53–0.80), with reported rural Australian origin compared with no such background aRR 1.49; 95% CI 1.22–1.83), and with reported previous personal experience with Indigenous people compared with none (aRR 1.40; 95% CI 1.14–1.72), but not with age group, qualification level or year of placement.

**Table 3:** Predictors of a student reporting feeling either ‘Confident’ or ‘Very confident’ at placement commencement

	**Crude**		**Adjusted**	
	**Relative risk (95% CI)**	***p***	**Relative risk (95% CI)**	***p***
**Age group at registration**				
< 23 years	Reference		Reference	
≥ 23 years	1.08 (0.88–1.34)	0.464	1.07 (0.87–1.31)	0.546
**Gender**^**a**^				
Male	Reference		Reference	
Female	0.71 (0.58–0.87)	0.001	0.65 (0.53–0.80)	< 0.001
**Rural Australian origin**				
No	Reference		Reference	
Yes	1.53 (1.24–1.88)	< 0.001	1.49 (1.22–1.83)	< 0.001
**Qualification level**				
Undergraduate	Reference		Reference	
Postgraduate	0.95 (0.76–1.20)	0.687	0.95 (0.76–1.19)	0.644
**Previous Indigenous experience**				
No	Reference		Reference	
Yes	1.43 (1.17–1.76)	0.001	1.40 (1.14–1.72)	0.001
**Placement year**				
2019	Reference		Reference	
2020	0.94 (0.71–1.24)	0.650	0.95 (0.73–1.25)	0.725
2021	0.87 (0.67–1.13)	0.284	0.82 (0.64–1.07)	0.139
2022	1.03 (0.73–1.46)	0.847	0.98 (0.71–1.36)	0.926

^**a**^No student identified their gender as ‘Other’

*CI* confidence interval

The proportion of students who reported feeling ‘Confident’ or ‘Very confident’ working with Aboriginal people increased from 41.9% to 82.0% between the commencement and end of placement (Fig. 2).

The baseline characteristics of 814 students who completed the commencement and the subgroup of 489 whose records comprised the principal analysis were similar in all regards except that attrition in survey completion between commencement and end of placement was notably more marked in 2022 compared with the other three years of the study (Table S1).

In the principal robust Poisson regression model (*N* = 489 placements; Table 4), increased confidence working with Indigenous people between the commencement and end of placement was independently associated with several baseline characteristics of the students as well as with certain attributes of their placements. Students identifying as female compared with males were more likely to report increased confidence (adjusted relative risk [aRR] 1.29; 95% confidence interval [95% CI] 1.07–1.55), but no association with students’ age was evident. Students from a background in rural Australia were less likely than other students to report increased confidence (aRR 0.77; 95% CI 0.63–0.94); those who reported having experience with Indigenous people prior to the placement had a lower likelihood of reporting increased confidence than those without previous experience (aRR 0.82; 95% CI 0.69–0.97). The placement attribute associated most strongly with increased confidence was interaction with Indigenous people within the placement setting (aRR 2.32; 95% CI 1.24–4.34). Students whose placement model was categorised as Blended/ Comprehensive had a higher likelihood of increased confidence than those who placement was categorised as Assisted/Supported (aRR 1.18, 95% CI 1.02–1.37). Placement length was also independently associated with increased confidence in a linear fashion (aRR for each additional day: 1.002; 95% CI 1.001–1.004). Compared with the pre-COVID pandemic year of 2019, increased confidence did not change significantly in any of the three subsequent years of the study period.

**Table 4:** Determinants of increased confidence dealing with Indigenous people during placements (*N* = 489)

	**Crude**		**Adjusted**	
	**Relative risk (95% CI)**	***p***	**Relative risk (95% CI)**	***p***
**Age group at registration**				
< 23 years	Reference		Reference	
≥ 23 years	0.96 (0.83–1.11)	0.564	1.00 (0.86–1.16)	0.996
**Gender**^**a**^				
Male	Reference		Reference	
Female	1.30 (1.07–1.57)	0.007	1.29 (1.07–1.55)	0.006
**Rural Australian origin**				
No	Reference		Reference	
Yes	0.81 (0.66–1.00)	0.049	0.77 (0.63–0.94)	0.011
**Qualification level**				
Undergraduate	Reference		Reference	
Postgraduate	0.95 (0.81–1.12)	0.532	0.98 (0.83–1.15)	0.767
**Previous Indigenous experience**				
No	Reference		Reference	
Yes	0.84 (0.71–0.98)	0.031	0.82 (0.69–0.97)	0.019
**Placement model**				
Assisted/Supported	Reference		Reference	
Blended/Comprehensive	1.25 (1.08–1.45)	0.003	1.18 (1.02–1.37)	0.031
**Indigenous interaction within placement**				
No	Reference			
Yes	2.35 (1.23–4.46)	0.009	2.32 (1.24–4.34)	0.008
**Indigenous interaction (external) during placement**				
No	Reference		Reference	
Yes	1.03 (0.87–1.20)	0.759	1.04 (0.89–1.22)	0.621
**Placement length (days)**^**b**^				
10-day minimum	Reference		Reference	
Per additional day	1.003 (1.002–1.005)	< 0.001	1.002 (1.001–1.004)	0.009
**Placement year**				
2019	Reference		Reference	
2020	0.95 (0.76–1.18)	0.658	0.88 (0.71–1.09)	0.233
2021	1.17 (0.98–1.40)	0.086	1.15 (0.97–1.38)	0.115
2022	0.88 (0.65–1.20)	0.415	0.89 (0.67–1.19)	0.441

^a^No student identified their gender as ‘Other’

^b^Modelled as a continuous variable; CI to third decimal place displayed for clarity

*CI* confidence interval

The sensitivity analysis (Table 5) excluded seventeen students (3.5%) who had reported the highest possible level of confidence in their commencement survey, for the purpose of investigating whether the results were robust to the influence of a ceiling effect. The adjusted estimates in this model were similar to those in the principal analysis, albeit in general marginally closer to the null. In consequence, the association between increased confidence and previous Indigenous experience was no longer statistically significant (aRR 0.86, 95% CI 0.73–1.01), and the association between the outcome and rural Australian origin was of borderline significance (aRR 0.83, 95% CI 0.68–1.00).

**Table 5:** Sensitivity analysis: determinants of increased confidence dealing with Indigenous people during placements (*N* = 472)^a^

	**Crude**		**Adjusted**	
	**Relative risk (95% CI)**	***p***	**Relative risk (95% CI)**	***p***
**Age group at registration**				
< 23 years	Reference		Reference	
≥ 23 years	0.96 (0.83–1.11)	0.564	0.99 (0.85–1.14)	0.857
**Gender**^**b**^				
Male	Reference		Reference	
Female	1.30 (1.07–1.57)	0.007	1.26 (1.05–1.51)	0.013
**Rural Australian origin**				
No	Reference		Reference	
Yes	0.81 (0.66–1.00)	0.049	0.83 (0.68–1.00)	0.050
**Qualification level**				
Undergraduate	Reference		Reference	
Postgraduate	0.95 (0.81–1.12)	0.532	0.97 (0.83–1.14)	0.736
**Previous Indigenous experience**				
No	Reference		Reference	
Yes	0.84 (0.71–0.98)	0.031	0.86 (0.73–1.01)	0.068
**Placement model**				
Assisted/Supported	Reference		Reference	
Blended/Comprehensive	1.25 (1.08–1.45)	0.003	1.17 (1.01–1.36)	0.034
**Indigenous interaction within placement**				
No	Reference			
Yes	2.35 (1.23–4.46)	0.009	2.31 (1.24–4.29)	0.008
**Indigenous interaction (external) during placement**				
No	Reference		Reference	
Yes	1.03 (0.87–1.20)	0.759	1.04 (0.90–1.22)	0.583
**Placement length (days)**^**c**^				
10-day minimum	Reference		Reference	
Per additional day	1.003 (1.002–1.005)	< 0.001	1.002 (1.000–1.004)	0.047
**Placement year**				
2019	Reference		Reference	
2020	0.95 (0.76–1.18)	0.658	0.91 (0.74–1.12)	0.365
2021	1.17 (0.98–1.40)	0.086	1.15 (0.96–1.37)	0.119
2022	0.88 (0.65–1.20)	0.415	0.94 (0.71–1.23)	0.643

^a^Excludes 17 students with maximum confidence response at commencement

^b^No student identified their gender as ‘Other’

^c^Modelled as a continuous variable; CI to third decimal place displayed for clarity. *CI* confidence interval

A small proportion of students (*n *= 24; 4.9%) reported a diminished level of confidence at the end of placement compared with their responses to the commencement survey. These students tended to be those with higher confidence at commencement: they represented 4/17 (23.5%) who had been ‘Very confident’, 18 of 188 (9.6%) who had been ‘Confident’, and 2 of 203 (1.0%) who had been ‘Neutral’.

## Discussion

Based on the findings of this Western Australian study of diverse allied health students, a substantial increase in the proportion of students who self-report confidence in working with Indigenous people can be achieved during rural placements. The likelihood of this change is associated with several attributes of the placement as well as with characteristics of the students themselves. The most influential determinant of increased confidence is inclusion within the placement of opportunities to interact directly with Indigenous people, which in this study was independently associated with a doubled frequency of increased confidence compared with placements that involved no such interaction. Students whose placement was categorised as ‘comprehensive/blended’, indicating a high level of contact with WACRH staff, had a higher likelihood of increased confidence than those who did not. Notably, WACRH employed a number of Aboriginal staff (ranging from two to seven at any one time) throughout the study period; two of these have been employed by WACRH for over ten years. Although these staff have generally not had clinical backgrounds, several have provided direct input into student placements, particularly in relation to cultural orientation and ‘clinical yarning’. WACRH staff also have ongoing opportunities to learn from Indigenous staff colleagues. Among female compared with male students, confidence working with Indigenous people at the commencement of a rural placement caught up disproportionately during the placement.

The findings of this study complement those of previously published qualitative research on the various aspects of placements that foster attitudes and skills that promote cultural safety among students of the health professions. From a broad international perspective, development of cultural competence during clinical placements is understood to be an individually varying process dependent on characteristics of both the placement and the student, through the four interrelated pathways of immersion in suitable healthcare environments, opportunities for interpersonal interaction while in these environments, observation by students of the cultural practices of others, and reflection by the student [24]. It has been recognised that in Australia, as in similar countries with a history of colonisation of Indigenous peoples such as New Zealand and Canada, universities have a responsibility to provide students of the health professions with teaching and learning that engenders culturally safe practice. This can be achieved through curricula that incorporate ‘Indigenous ways of knowing, being and doing into the curricula, understanding the local Indigenous histories and contexts, the adoption of online cultural education modules, and clinical placement partnerships with local Indigenous communities’ [25] [p110]. Although the influences on development of cultural competence among health professionals and students remain uncertain [24], there is evidence that various components of curriculum and student experience contribute, including single workshops [26] and single compulsory teaching units [27], as well as immersive service learning in an Indigenous community [28, 29].

Placement length is important in enabling students to experience a wider variety of sites and interactions with local people. The importance of increasing placement length to enhance students’ experiences and understandings needs to be emphasised while also recognising that it is often determined by specific professional accreditation requirements and therefore not determined by rurally based academics working to build the rural workforce. However, the length of WACRH placements—and to some extent the type of placement—is substantially dependent on the students’ discipline of study. For example, placements of speech pathology and social work students often extend across ≥ 14 weeks, whereas chiropractic and physiotherapy placements are always two- and five-weeks respectively. Given the large number of different disciplines (sixteen, some with very small student numbers) and the associations of discipline with placement type and length, the multivariable regression estimates were potentially biased when discipline was included as a covariate during preliminary analysis.

The importance of the findings depends on how self-reported confidence in the immediate term translates into long term attitudes and proficiency of performance. While the current study does not incorporate longitudinal follow-up data to address the durability of attitudinal changes associated with student placement, qualitative findings from other research on the outcomes of WACRH placements indicate that the personal and professional development legacy of placements can be enduring, at least in some students [30]. There is also evidence that at least for some health students who during their training become aware of the issues of Indigenous people related to life adversity and racism, this experience continues to inform their delivery of care and advocacy for better healthcare systems [31]. Ultimately, the attitudes and skills of individual health professionals are only one element of culturally safe healthcare for Indigenous people, which depends also attention to the voices of Indigenous people and their inclusion within health workforce and sustained support for culturally safe care across healthcare organisations and systems [32]. It was not possible in this study to investigate overconfidence and its consequences for culturally safe practice. However, the decrease in confidence at the end of the placement reported by a minority of students suggests that these individuals may have considered their confidence level at the outset to have been somewhat misplaced.

The baseline findings in relation to the demographic attributes of students are largely intuitive, insofar as prior direct experience with Indigenous people and an upbringing in rural Australia more broadly are predictably determinants of familiarity with Indigenous issues. The diminished confidence of female compared with male students at baseline is less straightforward, as there is some evidence that female health students report globally less confidence across diverse domains of performance including those interpersonal and communication skills [33], while there are conflicting findings that female students have greater confidence than males in relation to interpersonal matters [34].

### Strengths and limitations

This study was strengthened by the relatively large size of the overall sample, with the generalisability of findings enhanced by the demographic and professional diversity of the students. Limitations of the study are the inherently subjective nature of self-reported confidence as a proxy of a student’s proficiency in the care Indigenous people, the potential sensitivity of results to statistical modelling strategies, e.g., biasing of confidence increment estimates due to a ceiling effect (although this was minor as far as could be ascertained) and unmeasured confounders (such as those associated with discipline of study). the uncertainty of long-term consequences for the students’ future work choices and performance in relation to caring for Indigenous Australian people. The incomplete participation of eligible students in the surveys, particularly the considerable sample attrition between the commencement and end of placement in this regard, is a source of potential bias in ascertainment of students’ perspectives although no systematic disparity in completion surveys by baseline characteristics was evident.

## Conclusions

The findings of this study of a demographically and educationally diverse group of allied health students undertaking a rural placement suggest that a gain in confidence working with Indigenous Australian people during the placement is influenced both by attributes of the students themselves and by features of the placement. Importantly, the opportunity to interact with Indigenous people during the course of the placement is the key attribute of placements that enhances gain in confidence. Longer placements, along with structured support, allow more opportunities for interaction and engagement with Indigenous people. Future studies need to address the long-term implications of this increased confidence in relation to development of cultural competence and rural intention among health professional graduates, and ultimately for the impact on health outcomes among Indigenous Australian people.

## Supplementary Information

Supplementary Material 1.

## Data Availability

The dataset analysed for the current study is not publicly available due to confidentiality considerations, but the corresponding author can be contacted with requests related to data access.

## Abbreviations

aRR: Adjusted relative risk
CI: Confidence interval
WACRH: Western Australian Centre for Rural Health

## References

[1] Briggs D. Challenges for Health Systems: Australian Perspectives. Public Adm Policy. 2017;20:6–17.

[2] Dixit SK, Sambasivan M. A review of the Australian healthcare system: a policy perspective. SAGE Open Med. 2018;6:2050312118769211. 29686869 10.1177/2050312118769211 PMC5900819

[3] McGrail MR, Humphreys JS. Spatial access disparities to primary health care in rural and remote Australia. Geospat Health. 2015;10:138–43. 10.4081/gh.2015.358 26618314

[4] Wakerman J, Humphreys J, Russell D, Guthridge S, Bourke L, Dunbar T, et al. Remote health workforce turnover and retention: What are the policy and practice priorities? Hum Resour Health. 2019;17:99. 31842946 10.1186/s12960-019-0432-y PMC6915930

[5] Australian Institute of Health and Welfare. Indigenous health and wellbeing. Commonwealth of Australia. 2022. https://www.aihw.gov.au/reports/australias-health/indigenous-health-and-wellbeing. Accessed 1 Mar 2024.

[6] Australian Bureau of Statistics. Australian Aboriginal and Torres Strait Islander life expectancy. Commonwealth of Australia. 2023. https://www.abs.gov.au/statistics/people/aboriginal-and-torres-strait-islander-peoples/aboriginal-and-torres-strait-islander-life-expectancy/latest-release. Accessed 1 Mar 2024.

[7] Mitrou F, Cooke M, Lawrence D, Povah D, Mobilia E, Guimond E, et al. Gaps in Indigenous disadvantage not closing: a census cohort study of social determinants of health in Australia, Canada, and New Zealand from 1981–2006. BMC Public Health. 2014;14:201. 24568143 10.1186/1471-2458-14-201 PMC3937433

[8] Griffiths K, Coleman C, Lee V, Madden R. How colonisation determines social justice and Indigenous health—a review of the literature. J Popul Res. 2016;33:9–30.

[9] Menzies K. Understanding the Australian Aboriginal experience of collective, historical and intergenerational trauma. Int Soc Work. 2019;62:1522–34.

[10] Durey A, Thompson SC. Reducing the health disparities of Indigenous Australians: time to change focus. BMC Health Serv Res. 2012;12:151. 22682494 10.1186/1472-6963-12-151 PMC3431273

[11] Durey A, Haigh M, Katzenellenbogen JM. What role can the rural pipeline play in the recruitment and retention of rural allied health professionals? Rural Remote Health. 2015;15:1–11. 26290155

[12] Brown L, Smith T, Wakely L, Wolfgang R, Little A, Burrows J. Longitudinal tracking of workplace outcomes for undergraduate allied health students undertaking placements in Rural Australia. J Allied Health. 2017;46:79–87. 28561864

[13] Moran A, Nancarrow S, Cosgrave C, Griffith A, Memery R. What works, why and how? A scoping review and logic model of rural clinical placements for allied health students. BMC Health Serv Res. 2020;20:866. 32928199 10.1186/s12913-020-05669-6 PMC7489211

[14] Skinner TC, Semmens L, Versace V, Bish M, Skinner IK. Does undertaking rural placements add to place of origin as a predictor of where health graduates work? Aust J Rural Health. 2022;30:529–35. 35324046 10.1111/ajr.12864 PMC9545767

[15] Thomas J, Butler S, Battye K, Sefton C, Smith J, Skinner I, et al. Rural placements during undergraduate training promote future rural work by nurses, midwives and allied health professionals. Aust J Rural Health. 2021;29:253–8. 33982846 10.1111/ajr.12728

[16] Playford D, Moran MC, Thompson S. Factors associated with rural work for nursing and allied health graduates 15–17 years after an undergraduate rural placement through the University Department of Rural Health program. Rural Remote Health. 2020;20:5334. 32000498 10.22605/RRH5334

[17] Humphreys J, Lyle D, Barlow V. University departments of rural health: Is a national network of multidisciplinary academic departments in Australia making a difference? Rural Remote Health. 2018;18:4315.

[18] Walsh SM, Versace VL, Thompson SC, Browne LJ, Knight S, Lyle DM, et al. Supporting nursing and allied health student placements in rural and remote Australia: a narrative review of publications by university departments of rural health. Med J Aust. 2023;219:S14–9. 37544003 10.5694/mja2.52032

[19] Hardy BJ, Filipenko S, Smylie D, Ziegler C, Smylie J. Systematic review of Indigenous cultural safety training interventions for healthcare professionals in Australia, Canada, New Zealand and the United States. BMJ Open. 2023;13:e073320. 10.1136/bmjopen-2023-073320 PMC10551980 37793931

[20] Gray M, Thomas Y, Bonassi M, Elston J, Tapia G. Cultural safety training for allied health students in Australia. Aust J Indig Educ. 2021;50:274–83.

[21] Fullerton AS. A conceptual framework for ordered logistic regression models. Sociol Methods Res. 2009;38:306–47.

[22] Knol MJ, Le Cessie S, Algra A, Vandenbroucke JP, Groenwold RH. Overestimation of risk ratios by odds ratios in trials and cohort studies: alternatives to logistic regression. CMAJ. 2012;184:895–9. 22158397 10.1503/cmaj.101715 PMC3348192

[23] Chen W, Qian L, Shi J, Franklin M. Comparing performance between log-binomial and robust Poisson regression models for estimating risk ratios under model misspecification. BMC Med Res Methodol. 2018;18:63. 29929477 10.1186/s12874-018-0519-5 PMC6013902

[24] Liu J, Li S. An ethnographic investigation of medical students’ cultural competence development in clinical placements. Adv Health Sci Educ. 2023;28:705–39. 10.1007/s10459-022-10179-7 PMC10356648 36371573

[25] Anstice NS, Alam K, Armitage JA, Biles B, Black JM, Boon MY, et al. Developing culturally safe education practices in optometry schools across Australia and Aotearoa New Zealand. Clin Exp Optom. 2023;106:110–8. 36336833 10.1080/08164622.2022.2136514

[26] Durey A, Halkett G, Berg M, Lester L, Kickett M. Does one workshop on respecting cultural differences increase health professionals’ confidence to improve the care of Australian Aboriginal patients with cancer? An evaluation. BMC Health Serv Res. 2017;17:660. 28915810 10.1186/s12913-017-2599-z PMC5603013

[27] Flavell H, Thackrah R, Hoffman J. Developing Indigenous cultural competence: a model for implementing Indigenous content into curricula. J Teach Learning Grad Employab. 2013;4:39–63.

[28] Thackrah RD, Thompson SC, Durey A. “Listening to the silence quietly”: Investigating the value of cultural immersion and remote experiential learning in preparing midwifery students for clinical practice. BMC Res Notes. 2014;7:685. 25274179 10.1186/1756-0500-7-685 PMC4283110

[29] West M, Sadler S, Hawke F, Munteanu SE, Chuter V. Effect of a culturally safe student placement on students’ understanding of, and confidence with, providing culturally safe podiatry care. J Foot Ankle Res. 2021;14:9. 33499892 10.1186/s13047-021-00450-2 PMC7836510

[30] Thackrah RD, Thompson SC. Learning from follow-up of student placements in a remote community: a small qualitative study highlights personal and workforce benefits and opportunities. BMC Med Educ. 2019;19:331. 31484513 10.1186/s12909-019-1751-3 PMC6727324

[31] Thackrah RD, Wood J, Thompson SC. Longitudinal follow up of early career midwives: Insights related to racism show the need for increased commitment to cultural safety in aboriginal maternity care. Int J Environ Res Public Health. 2021;18:1–14. 10.3390/ijerph18031276 PMC7908636 33572624

[32] De Zilva S, Walker T, Palermo C, Brimblecombe J. Culturally safe health care practice for Indigenous Peoples in Australia: A systematic meta-ethnographic review. J Health Serv Res Policy. 2022;27:74–84. 34875923 10.1177/13558196211041835

[33] Vajapey SP, Weber KL, Samora JB. Confidence gap between men and women in medicine: a systematic review. Curr Orthop Pract. 2020;31:494–502.

[34] Papyrina V, Strebel J, Robertson B. The student confidence gap: Gender differences in job skill self-efficacy. J Educ Bus. 2021;96:89–98.

