# Supplementary material for: Influences on improved confidence among allied health students in working with Australian Indigenous people during a rural placement: a pre-post survey study
Source: BMC Med Educ. 2024 Nov 1;24:1246. doi: 10.1186/s12909-024-06207-2 (PMC11529035; doi:10.1186/s12909-024-06207-2)
Supplement: Supplementary file 1 — Supplementary Material 1. [file 12909_2024_6207_MOESM1_ESM.docx]

**Table S1.** Comparative baseline characteristics of (i) all students who participated in the commencement-of-placement questionnaire (N=814), and (ii) the subgroup who participated in both commencement and end questionnaires, and provided paired commencement/end-of-placement data on confidence working with Aboriginal people (N=489)

|  | 1. **All students who participated in commencement questionnaire** | | |  | 1. **Students who provided paired commencement-end data on confidence working with Aboriginal people** | | |
| --- | --- | --- | --- | --- | --- | --- | --- |
|  | **Range Median** | **Mean** | **SD** |  | **Range Median** | **Mean** | **SD** |
| **Age (years)** | 18–60 23 | 25.0 | 5.6 |  | 18–60 23 | 25.1 | 6.0 |
|  |  |  |  |  |  |  |  |
|  |  | **n** | **%** |  |  | **n** | **%** |
| **Gender** | Female | 573 | 70.4 |  | Female | 349 | 71.4 |
|  | Male | 241 | 29.6 |  | Male | 140 | 28.6 |
|  | Other | 0 | 0.0 |  | Other | 0 | 0.0 |
|  |  |  |  |  |  |  |  |
| **Rural origin** | No | 637 | 78.2 |  | No | 375 | 76.7 |
|  | Yes (Aust) | 165 | 20.3 |  | Yes (Australia) | 108 | 22.1 |
|  | Yes (Overseas) | 12 | 1.5 |  | Yes (Overseas) | 6 | 1.2 |
|  |  |  |  |  |  |  |  |
| **Qualification Level** | Undergrad | 560 | 68.8 |  | Undergraduate | 329 | 67.3 |
|  | Postgrad | 254 | 31.2 |  | Postgraduate | 160 | 32.7 |
|  |  |  |  |  |  |  |  |
| **Prior Indigenous experience** | No | 534 | 65.6 |  | No | 323 | 66.1 |
|  | Yes | 280 | 34.4 |  | Yes | 166 | 33.9 |
|  |  |  |  |  |  |  |  |
| **Placement model** | Assisted/Supported | 460 | 56.5 |  | Assisted/Supported | 272 | 55.6 |
|  | Comprehensive/Blended | 354 | 43.5 |  | Comprehensive/Blended | 217 | 44.4 |
|  |  |  |  |  |  |  |  |
| **Placement year** | 2019 | 205 | 25.1 |  | 2019 | 139 | 28.4 |
|  | 2020 | 190 | 23.3 |  | 2020 | 122 | 25.0 |
|  | 2021 | 229 | 28.1 |  | 2021 | 176 | 36.0 |
|  | 2022 | 190 | 23.3 |  | 2022 | 52 | 10.6 |
|  |  |  |  |  |  |  |  |
| **Discipline** | Audiology | 16 | 2.0 |  | Audiology | 11 | 2.3 |
|  | Biomed/Med Science | <5 | <1.0 |  | Biomed/Med Science | <5 | <1.0 |
|  | Chiropractic | 145 | 17.8 |  | Chiropractic | 66 | 13.5 |
|  | Dietetics/Nutrition | 26 | 3.2 |  | Dietetics/Nutrition | 14 | 2.9 |
|  | Exercise Physiology | 69 | 8.5 |  | Exercise Physiology | 38 | 7.8 |
|  | Health Promotion | 17 | 2.1 |  | Health Promotion | 13 | 2.7 |
|  | Health Science | <5 | <1.0 |  | Health Science | <5 | <1.0 |
|  | Medical Imaging | 25 | 3.1 |  | Medical Imaging | 15 | 3.1 |
|  | Music Therapy | <5 | <1.0 |  | Music Therapy | <5 | <1.0 |
|  | Occupational Therapy | 85 | 10.4 |  | Occupational Therapy | 58 | 11.9 |
|  | Pharmacy | 138 | 17.0 |  | Pharmacy | 94 | 19.2 |
|  | Physiotherapy | 121 | 14.9 |  | Physiotherapy | 65 | 13.3 |
|  | Podiatry | 33 | 4.1 |  | Podiatry | 24 | 4.9 |
|  | Public Health | 10 | 1.2 |  | Public Health | 6 | 1.2 |
|  | Social Work | 89 | 10.9 |  | Social Work | 59 | 12.1 |
|  | Speech Pathology | 34 | 4.2 |  | Speech Pathology | 22 | 4.5 |

Cell numbers <5 obscured to protect confidentiality
